# Characterization of oxylipins and dioxygenase genes in the asexual fungus *Aspergillus niger*

**DOI:** 10.1186/1471-2180-9-59

**Published:** 2009-03-23

**Authors:** Mayken W Wadman, Ronald P de Vries, Stefanie IC Kalkhove, Gerrit A Veldink, Johannes FG Vliegenthart

**Affiliations:** 1 Bioorganic Chemistry, Utrecht University, 3584 CH, Utrecht, the Netherlands; 2 Microbiology, Utrecht University, 3584 CH, Utrecht, the Netherlands

## Abstract

**Background:**

*Aspergillus niger *is an ascomycetous fungus that is known to reproduce through asexual spores, only. Interestingly, recent genome analysis of *A. niger *has revealed the presence of a full complement of functional genes related to sexual reproduction [1]. An example of such genes are the dioxygenase genes which in *Aspergillus nidulans*, have been shown to be connected to oxylipin production and regulation of both sexual and asexual sporulation [2-4]. Nevertheless, the presence of sex related genes alone does not confirm sexual sporulation in *A. niger*.

**Results:**

The current study shows experimentally that *A. niger *produces the oxylipins 8,11-dihydroxy octadecadienoic acid (8,11-diHOD), 5,8-dihydroxy octadecadienoic acid (5,8-diHOD), lactonized 5,8-diHOD, 8-hydroxy octadecadienoic acid (8-HOD), 10-hydroxy octadecadienoic acid (10-HOD), small amounts of 8-hydroxy octadecamonoenoic acid (8-HOM), 9-hydroxy octadecadienoic acid (9-HOD) and 13-hydroxy octadecadienoic acid (13-HOD). Importantly, this study shows that the *A. niger *genome contains three putative dioxygenase genes, *ppoA*, *ppoC *and *ppoD*. Expression analysis confirmed that all three genes are indeed expressed under the conditions tested.

**Conclusion:**

*A. niger *produces the same oxylipins and has similar dioxygenase genes as *A. nidulans*. Their presence could point towards the existence of sexual reproduction in *A. niger *or a broader role for the gene products in physiology, than just sexual development.

## Background

The fungal kingdom comprises a large group of organisms (estimated to consist of over 1.5 million species) with only 5% identified thus far. Fungal species can survive in virtually all biotopes on earth, as they have been identified in water and soil, and on plants and animals. Part of their success comes from the ability to use different reproductive strategies, which provide increased flexibility for diverse environmental requirements. Fungal species can produce sexual cells and/or asexual cells in distinct reproductive structures. Some fungi are able to reproduce both sexually and asexually depending on the circumstances, while others display one mode of reproduction, only. Sexual reproduction and recombination allows the repair of naturally occurring mutations and results in new genotypes and phenotypes that allow for natural selection [5]. On the other hand, asexual reproduction provides the ability to disperse numerous genetically identical mitospores, without the metabolic costs of sexual reproduction [5].

*Aspergillus niger *is an ascomycetous fungus that is considered to reproduce through asexual spores, only. Since *A. niger *is used as a host for the production of homologous and heterologous proteins and commercially important compounds (such as citric acid), the potential presence of a sexual cycle is highly significant for strain improvement. Recent analysis of the *A. niger *genome has revealed the presence of a full complement of genes related to sexual reproduction [1]. It was therefore suggested that there could be a latent sexual potential in *A. niger*. A similar observation applies to *Aspergillus fumigatus *and *Aspergillus oryzae*, both only known to reproduce asexually, so far. Comparison of the two genomes to the genome of *Aspergillus nidulans *(please note that the holomorph is correctly named *Emericella nidulans*, but is hereafter mentioned as *A. nidulans*), which has a known sexual cycle, suggests that both *A. fumigatus *and *A. oryzae *may be capable of sexual reproduction [6]. It has yet to be determined whether genes related to sexual reproduction in supposedly asexual fungi are functional.

Dioxygenase genes with homology to mammalian prostaglandin synthase (PGS) have been connected to the formation of oxylipins in *A. nidulans*. Dioxygenase genes and oxylipins are linked to reproduction as they regulate the balance between sexual and asexual sporulation [2-4]. The goal of this study was to investigate whether or not oxylipins and dioxygenase genes related to sexual reproduction are also present in the asexual fungus *A. niger*.

## Results

### RP-HPLC analysis

**Figure 1:**
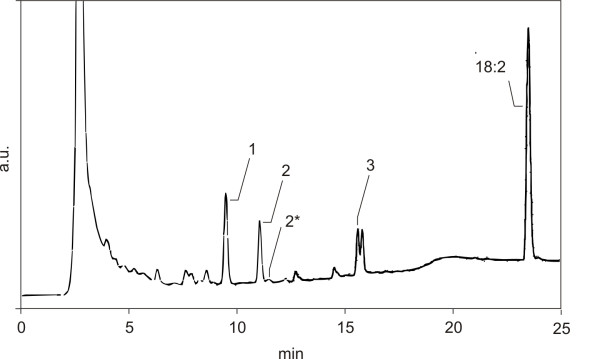
**RP-HPLC chromatogram (λ = 200 nm) of the reaction of a crude extract of *A. niger *N402 biomass with 18:2**. Indicated are peak 1 (9.2 min; 8,11-diHOD), peak 2 (10,8 min; 5,8-diHOD), peak 2* (10.9 min, λ_max _218 nm; lactonized 5,8-diHOD), and peak 3 (15.1 min; 8-HOD), the major fatty acid metabolites. RP-HPLC analysis and purification of the fatty acid products were carried out on a Cosmosil 5C18-AR (5 μm; 250 × 4.6 mm i.d.; Nacalai Tesque, Kyoto, Japan) reversed-phase column using a gradient system (solvent A: methanol/water/acetic acid (50:50:0.01, v/v/v); solvent B: methanol/water/acetic acid (95:5:0.01, v/v/v)) with the following gradient program: 45% solvent A for 1 min, followed by a linear increase of solvent B up to 100% within 10 min and finally an isocratic post-run at 100% solvent B for 10 min. The flow-rate was 1 mL/min. Reference compounds of dihydroxy fatty acids had a retention time of 9–11 min, whereas monohydroxy fatty acid references eluted between 15–18 min.

A crude extract of *A. niger *N402 biomass was incubated with 18:2 and the reaction mixture was extracted with SPE and analyzed on RP-HPLC. A typical HPLC chromatogram is shown in Fig. 1. Incubation with 18:2 resulted in the appearance of three large peaks in the HPLC chromatogram and a smaller one. Similar results were obtained for *A. niger *UU-A049.1, A. niger Δ*ppoA *(UU-A050.3), *A. niger *Δ*ppoD *(UU-A051.26) and *A. nidulans *WG096 (data not shown). For each strain, fatty acid reaction products were fractionated on HPLC and after derivatization further investigated with GC/MS. Structures of oxygenated fatty acids were deduced from the spectra of the TMS ethers of methyl ester derivatives.

### GC/MS analysis of dihydroxy fatty acids (RP-HPLC peak 1, peak 2 and peak 2*)

Hydrogenated dihydroxy fatty acids as TMS ethers of methyl ester derivatives from RP-HPLC peak 1 (Fig. 1) were separated on GC and one dominant peak was present in the chromatogram. The mass spectrum was similar that of the TMS ether of methyl 8,11-dihydroxy octadecanoate [7]. The GC retention time and mass spectrum of the non-hydrogenated sample and the GC retention time and mass spectrum of TMS ether of methyl 8,11-dihydroxy-9,12-octadecadienoate showed that the major fatty acid product in RP-HPLC peak 1 (Fig. 1) was 8,11-dihydroxy octadecadienoic acid (8,11-diHOD) [7].

Hydrogenated RP-HPLC peak 2 (Fig. 1) as TMS ether of methyl ester derivative was separated on GC and one dominant peak was present in the chromatogram. The mass spectrum showed characteristic peaks stemming from cleavages around the two oxygenated C atoms that indicated the presence of TMS ether of methyl 5,8-dihydroxy octadecanoate. Comparison of the mass spectrum from hydrogenated and non-hydrogenated samples showed that the TMS ether of methyl 5,8-dihydroxy octadecanoate was derived from the TMS ether of methyl 5,8-dihydroxy-9,12-octadecadienoate. This was evidenced by the molecular ion at *m/z *470 and by the characteristic fragments resulting from cleavage around the double bonds and oxygenated C atoms [8]. Thus RP-HPLC peak 2 (Fig. 1) proved to be 5,8-diHOD.

RP-HPLC peak 2* was analyzed as a part of RP-HPLC peak 2, due to overlap. Hydrogenation of the TMS ether derivative showed peaks stemming from cleavage around an oxygenated C-atom. The molecular ion at *m/z *370 evidenced that this compound was TMS ether of lactonized 5,8-dihydroxyoctadecanoate. Comparing the hydrogenated sample with the non-hydrogenated sample showed that TMS ether of lactonized 5,8-dihydroxy octadecanoate probably originated from lactonized 5,8-diHOD.

### GC/MS analysis of monohydroxy fatty acids (RP-HPLC peak 3)

In the GC chromatogram of the hydrogenated monohydroxy fatty acids of RP-HPLC peak 3 (Fig. 1) as TMS ethers of methyl ester derivatives, one prominent peak was present. The mass spectrum identified it as a mixture of the TMS ethers of methyl 8-hydroxy octadecanoate, methyl 10-hydroxy octadecanoate and a small amount of methyl 9-hydroxy octadecanoate. Also, a small peak of methyl 13-hydroxy octadecanoate was present in the GC chromatogram. In the GC/MS analysis of the corresponding non-hydrogenated monohydroxy fatty acids as TMS ethers of methyl ester derivatives, three peaks were visible in the GC chromatogram. Reference compounds indicated that GC peak 1 (18.3 min) was TMS ether of methyl 8-hydroxy octadecadienoate because of the fragmentation pattern and retention time of the non-hydrogenated sample [7]. The mass spectrum of TMS ether of methyl 10-hydroxy octadecanoate, GC peak 2 (18.4 min), showed that this compound originated from 10-hydroxy octadecadienoic acid (10-HOD). The mass spectrum of GC peak 4 (19.1 min) and the mass spectra of reference compounds showed that TMS ethers of methyl 13-hydroxy octadecanoate and methyl 9-hydroxy decanoate were derived from 13-hydroxy octadecadienoic acid (13-HOD) and 9-hydroxy octadecadienoic acid (9-HOD), respectively. Thus, RP-HPLC peak 3 (Fig. 1) was composed of 8-HOD (20), 10-HOD (18), 13-HOD (1) and 9-HOD (1).

GC/MS analysis of monohydroxy fatty acids eluting after RP-HPLC peak 3 (Fig. 1) as TMS ethers of methyl ester derivatives showed that a small amount of 8-HOM was also present (data not shown).

### Characteristics of oxylipin formation

Incubation with [U-^13^C] 18:2 showed that all oxygenated fatty acid products (RP-HPLC peak 1 to peak 3, Fig. 1) represented a mixture of converted 18:2 from endogenous and exogenous sources. The conversion of 500 nmol exogenously supplied 18:2 was about 50% of the total conversion, as judged by the ratio of [U-^13^C] labeled fragments to unlabeled fragments on GC/MS. In a reaction with 20:4 was shown that 20:4 it was not converted by an *A. niger *N402 crude extract.

### Endogenous oxylipins

Endogenous oxylipins of *A. niger *N402 biomass were extracted and analyzed on GC/MS. Oxylipin levels were very low when compared to the total ion-current of the internal standard 17:0. Traces of 5,8-diHOD, 8,11-diHOD, 8-HOD, 10-HOD, 13-HOD and 8-HOM were detected, however, oxylipin levels were generally just above background. Similar results were obtained for *A. niger *UU-A049.1, *A. niger ΔppoA *(UU-A050.3), *A. niger ΔppoD *(UU-A051.26) and *A*.*nidulans *WG096.

### Identification of three putative *A. niger *dioxygenase genes, *ppoA*, *ppoC *and *ppoD*

A search of the *A. niger *N402 genomic database identified three putative dioxygenase genes *ppoA*, *ppoC *and *ppoD *that are located on chromosomes 6, 4 and 3, respectively, and contained 6, 12, and 11 introns, respectively. The deduced amino acid sequences of PpoA (1080 aa, 120 kD), PpoC (1110 aa, 125 kD) and PpoD (1164 aa, 131 kD) represented proteins with strong homology to *G. graminis *LDS. *A. niger *PpoA and PpoC were closely related to *A. nidulans *PpoA and PpoC (Table 1). Comparing the sequence of *A. niger *PpoD with those of PpoA, PpoB and PpoC from *A. nidulans *showed that *A. niger *ppoD had strongest similarity to *A. nidulans *PpoA and PpoC and not to *A. nidulans *PpoB (Table 1).

**Table 1:** Comparisson of predicted *A. niger *putative dioxygenases PpoA, PpoC and PpoD

Protein	Protein	*E*-value	Identities %	Positives %	Gaps %
*A. niger *PpoA	*A. nidulans *PpoA	0	69	81	7
					
	*A. nidulans *PpoC	0	37	56	10
	*A. nidulans *PpoB	1 × 10^-68^	43	53	21
	*G. graminis *LDS	0	45	60	8
*A. niger *PpoC	*A. nidulans *PpoC	0	60	75	10
					
	*A. nidulans *PpoA	0	47	64	10
	*A. nidulans *PpoB	8 × 10^-86^	39	51	20
	*G. graminis *LDS	3 × 10^-174^	41	58	10
*A. niger *PpoD	*A. nidulans *PpoA	5 × 10^-177^	38	55	11
					
	*A. nidulans *PpoC	8 × 10^-161^	31	46	12
	*A. nidulans *PpoB	5 × 10^-70^	41	52	19
	*G. graminis *LDS	1 × 10^-143^	38	55	2

**Figure 2:**
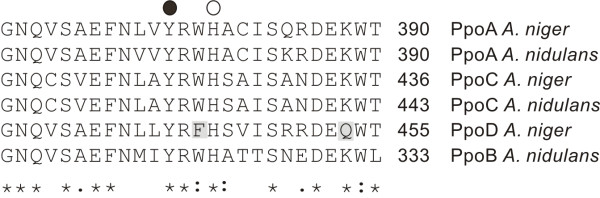
**Amino acid alignment of the predicted proximal His domain in *A. niger *PpoA, PpoC and PpoD to A. nidulans PpoA, PpoB and PpoC**. Identical amino acids are marked with asterisks; similar amino acids are marked with colons. The proximal His and the Tyr residue important for catalysis in PGS are marked with ○ and ● respectively. Deviating amino acids (Phe instead of Trp, and Gln instead of Lys), in *A. niger *PpoD are indicated in grey.

In analogy with *G. graminis *LDS and *A. nidulans *Ppo's, *A. niger *PpoA, PpoC and PpoD showed homology to animal PGS (*E*-values > 7 × 10^-21^; > 3 × 10^-24^; > 3 × 10^-18^, respectively). *A. niger *PpoA, PpoC and PpoD also contained the distal (202; 246; 265, respectively) and proximal (377; 424; 444, respectively) His, and Tyr (374; 420; 441, respectively) residues, essential for catalytic activity of PGS. Amino acid analysis of the predicted proximal His domain revealed that PpoD differed from the other *Aspergillus *Ppo's in having a Phe (443) instead of a Trp residue between the proximal His and Tyr residues and that a Lys, conserved in the other Ppo's, was replaced by a Gln (453) residue (Fig. 2)

**Figure 3:**
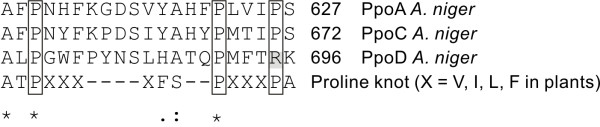
**Amino acid alignment of the predicted proline knot motif in *A. niger *PpoA, PpoC and PpoD to the proline knot motif in plants **[9,24]. Identical amino acids are marked with asterisks; similar amino acids are marked with colons. The conserved Pro residues are indicated with boxes. The third Pro residue in *A. niger *PpoD is replaced with an Arg residue, indicated in grey.

Putative *A. niger *PpoA and PpoC contained the proline knot motif that targets proteins to oil bodies in plants [4,9]. In contrast *A. niger *PpoD did not contain the proline knot motif, the third Pro residue was replaced by an Arg residue (Fig. 3).

### Phenotypic characterization of *A. niger *transformants

To study the connection of the *A. niger *putative dioxygenase genes to oxylipin formation and reproduction, *ppoA *and *ppoD *were inactivated by homologous recombination of the domain encoding the catalytic site with the *argB *cassette. *A. niger *Δ*ppoA *and Δ*ppoD *mutants had no alterations in radial growth. Also their response to osmotic, oxidative and temperature stress, and combinations thereof, did not differ from the reaction of the wild type. No effect on sporulation was observed for the *ppoA *and *ppoD *disruption strains or the *ppoA *multicopy strain. However, a 34% reduction in conidiospores was observed in the *ppoC *multicopy strain. In experiments where linoleic acid was added, all strains showed reduced conidiospore counts compared to the wild type.

### *A. niger *microarray analysis

**Figure 4:**
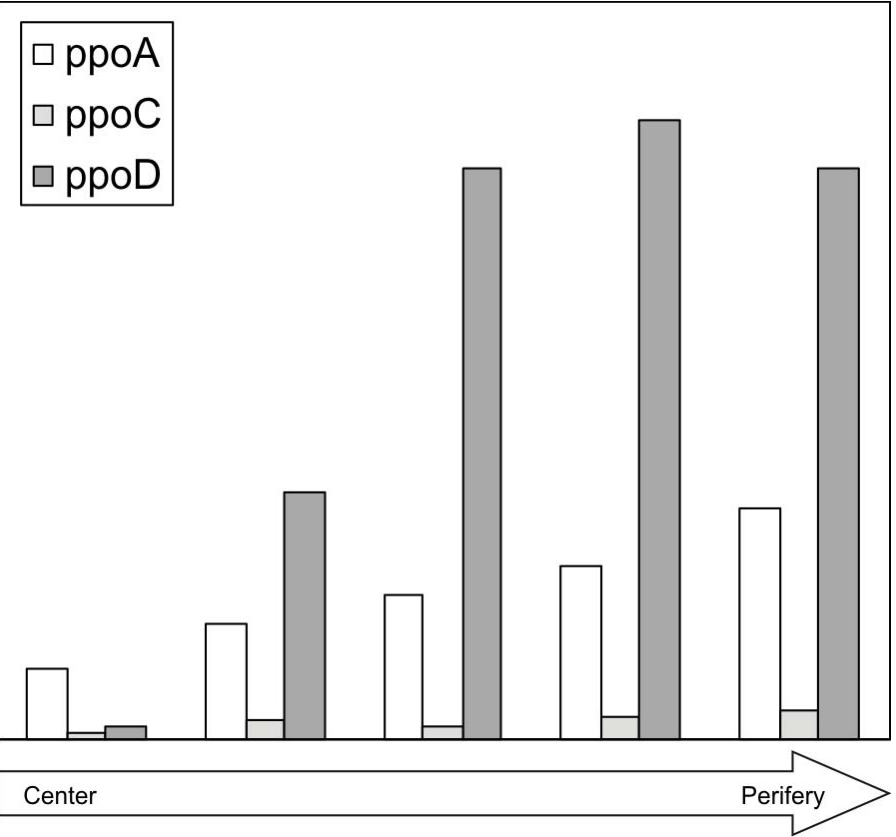
**Microarray analysis of expression levels of *A. niger *putative dioxygenase genes *ppoA*, *ppoC *and *ppoD *on maltose**. Five distinct zones were taken from the center to the perifery. Indicated are the relative expression levels.

Analysis of expression levels of *A. niger *putative dioxygenases *ppoA*, *ppoC *and *ppoD *showed that the three genes were expressed from the center to the periphery of the *A. niger *colonies grown on maltose, however, the level of expression differed (Fig. 4). The genes *ppoA *and *ppoD *were expressed mainly in the periphery, while levels of *ppoC *expression were equally distributed throughout the colony and low in comparison to the expression of *ppoA *and *ppoD*. Similar results were obtained during growth on D-xylose (data not shown). Since the *A. niger *strains were grown in sandwiched cultures, the formation of conidia was suppressed. Expression profiles of dioxygenase genes may be different in sporulating colonies.

## Discussion

The goal of this study was to investigate whether or not oxylipins and dioxygenase genes, that are related to both asexual and sexual reproduction, are present in the asexual fungus *A. niger*.

Using RP-HPLC and GC/MS, this study demonstrated that *A. niger *converted 18:2 mainly into 8,11-diHOD, 5,8-diHOD, lactonized 5,8-diHOD, 8-HOD and 10-HOD. The reaction with [U-^13^C] 18:2 showed that these compounds were produced from a mixture of exogenously added and endogenously present 18:2.

The presence of 5,8-diHOD, lactonized 5,8-diHOD, 8-HOD and 10-HOD, but not the presence of 8,11-diHOD were reported in *A. nidulans *[8,10-13] and related to sexual reproduction [2-4]. Interestingly, in the present study 8,11-diHOD was one of the oxylipins formed by *A. nidulans*. During the preparation of this manuscript, a study was published showing that the asexual fungus *A. fumigatus *also produced 5,8-diHOD, 8,11-diHOD 8-HOD and 10-HOD [13]. This indicates that *A. niger*, *A. nidulans *and *A. fumigatus *all produce the same oxylipins.

Analysis of the *A. niger *genome revealed that this fungus contains three putative dioxygenase genes, *ppoA*, *ppoC *and *ppoD*. A *ppoB *homologue was not present. *A. niger *transformants lacking the *ppoA *or *ppoD *gene were not altered in their ability to produce oxylipins and sporulation. A reduction in conidiospore formation was observed in the *ppoC *multicopy strain. In contrast, in *A. nidulans ppoA*, *ppoB *or *ppoC *were found to be connected to oxylipin production and to modification of sexual and asexual sporulation. Deletion of *ppoA*, *ppoB *or *ppoC *was demonstrated to reduce the level of 8-HOD, 8-HOM and 8-HOM, respectively [2-4]. But a later study showed that deletion of *ppoA *led to a reduction of 8-HOD and 5,8-diHOD formation and that elimination of *ppoC *reduced 10-HOD formation [13]. The removal of *ppoB *did not alter oxylipin production [13]. In addition, deletion of *ppoA *or *ppoB *from the *A. nidulans *genome increased the ratio of asexual to sexual spores [3,4]. Elimination of *ppoC *on the other hand, significantly reduced the ratio of asexual to sexual spores [2]. Absence of a phenotype for the disruption strains of *A. niger *for *ppoA *and *ppoD*, could suggest that they are non-essential or that they in fact have the same function. Future studies into these genes should include construction of double-disruptants. The inability to isolate *ppoC *disruptants might suggest that this is an essential gene in *A. niger *even though this is not the case in *A. nidulans *[2] and could possibly indicate significant differences in the role of these genes in different fungi. When linoleic acid was added, all strains showed reduced asexual sporulation compared to the wild type, suggesting that addition of linoleic acid could not be compensated for when the production of the different Ppo's is altered in *A. niger*.

*A. niger *PpoD had deviating amino acid residues in the vicinity of the proximal His domain and did not contain the proline knot motif (Fig. 3). This motif targets plant proteins to oil bodies and it has been demonstrated that fungi target such proteins to oil bodies as well [14]. In addition, the proline knot is predicted to facilitate the formation of an antiparallel α-helix or β-strand [9]. Therefore, *A. niger *PpoD likely differs from the other Ppo's in its three dimensional structure It could be argued that the presence of *ppoD *instead of *ppoB *in *A. niger *is related to the reproductive differences between *A. niger *and *A. nidulans*. However, this seems unlikely since the genomes of *Aspergillus clavatus*, *Aspergillus oryzae *and *Aspergillus terreus*, all not known to reproduce sexually, do contain *ppoB *homologues. Additionally, *Histoplasma capsulatum*, a fungus belonging to the same class as the *Aspergilli*, contains *ppoD *and also does not contain *ppoB*, but is able to produce sexual spores [3]. Expression analysis of *A. niger ppoA*, *ppoC *and *ppoD *shows that these genes are expressed and their expression levels depend on the fungus' developmental stage (Fig. 4). It should be noted that *A. niger *is heterothallic and requires mating between two isolates with different mating types. Despite the fact that *A. niger *appears to contain all the genes required for a sexual cycle, until now, no sexual cycle has been observed for *A. niger *on any of a broad range of growth conditions (Paul Dyer, personal communication). In contrast, *A. nidulans *is a homothallic species in which both mating types are present in a single strain and can therefore cross with itself. This difference might hint towards different strategies for regulation of sexual and asexual development. Studies of these genes in other homothallic and heterothallic Aspergilli, could demonstrate whether this is a general difference between homothallic and heterothallic species. This could include the presence or absence of expression of specific dioxygenase genes.

Strictly asexual species are considered an evolutionary endpoint, and truly asexual species are thought to be extremely rare [5]. Sequencing of fungal genomes and comparative analysis of sexual and asexual species show that fungi that have long been considered asexual organisms, may have a latent potential for sexual reproduction [1,6]. Nevertheless, the presence of sex related genes alone, does not confirm sexual reproduction.

## Conclusion

This study shows that *A. niger *produces the same oxylipins and has similar dioxygenase genes as *A. nidulans*. Even though, the functionality of these genes remains as yet to be proven, their presence could point towards the existence of sexual reproduction in *A. niger *or a broader role for the gene products in physiology, than just sexual development.

## Methods

### Materials

All chemicals used were commercially obtained and of analytical grade. Linoleic acid (9*Z*,12*Z*-octadecadienoic acid, 18:2, 99% pure), arachidonic acid, 5*Z*,8*Z*,11*Z*,14*Z*-eicosatetraenoic acid, 20:4, 99% pure) and margaric acid (heptadecanoic acid, 17:0, 99% pure) were obtained from Sigma (St. Louis, MO). [U-^13^C] 18:2 (99% pure) was obtained from Isotec (Matheson Trigas, Irving, TX). Solutions of 30 mM fatty acid were stored in methanol under N_2 _at -20°C until use.

### Strains, media and culture conditions

*Aspergillus *strains used are listed in Table 2. Cultures were grown in minimal medium containing trace elements and 1% glucose as carbon source, unless otherwise indicated in the text [15]. Appropriate supplements (8 μM nicotinamide, 1.5 mM leucine, 5 mM uridine) were added to the media to complement auxotrophic mutations. *Aspergillus *cultures were inoculated with 10^6 ^spores/ml and grown at 30°C on a rotary shaker (Inova 2300; New Brunswick Scientific, Edison, NJ) at 250 rpm. For growth on solid media 1.5% of agar was added. Strains were grown in 25 ml of liquid medium in Petri dishes under stationary conditions at 30°C. Alternatively, strains were grown in 50 ml of liquid medium at 30°C in a rotary shaker at 250 rpm. Mycelial mats were collected after 72 h, dried between filter paper sheets and frozen in liquid nitrogen.

**Table 2:** *Aspergillus *strains used in this study

Strain	Genotype
*A. niger *N402 (FGSCA733)	*cspA*1
*A. niger *UU-A049.1	*nicA*1, *leuA*1, *pyrA*6, *Δarg*B:: *A. niger argB*
*A. niger ΔppoA *UU-A050.3	*nicA*1, *leuA*1, *pyrA*6, *Δarg*B:: *ppoA *disruption construct
*A. niger ΔppoD *UU-A051.26	*nicA*1, *leuA*1, *pyrA*6, *Δarg*B:: *ppoD *disruption construct
*A. nidulans *WG096 (FGSC187)	*pabaA*1, *yA*2

### Oxylipin characterization and analysis of enzymatic capacity

For analysis of endogenously present oxylipins, samples were lyophilized, weighed and homogenized mechanically using a microdismembrator (B. Braun GmbH, Melsungen, Germany). Free fatty acids and their derivatives were extracted with 80% methanol 1:10 (w/v), centrifuged at 4°C, 2500 × g for 20 min and recovered by solid phase extraction (SPE, Oasis HLB 200 mg; Waters, Milford, MA). 17:0 was used as an internal standard.

The enzymatic capacity to oxygenate fatty acids of *Aspergillus *strains was examined as follows. Samples were homogenized, extracted with phosphate buffer (50 mM sodium phosphate pH 6.5, 5:1 w/v) and centrifuged at 4°C, 2500 × g for 20 min. The supernatant (crude extract) was filtered through cheesecloth and used immediately. Typically, 4 mL phosphate buffer was mixed with 1 mL crude extract, rigorously stirred and incubated with 120 μM substrate for 30–45 min at room temperature under a continuous flow of O_2_. Fatty acids and reaction products were recovered directly by SPE.

### RP-HPLC and GC/MS analysis

SPE eluates were concentrated under N_2_, and analyzed by RP-HPLC. Analysis by GC/MS of the fatty acid products as TMS ethers of methyl ester derivatives was performed as described previously [16]. The fatty acid methylation reagent was diazomethane. For GC/MS analysis, samples were analyzed before and after hydrogenation. Oxylipins were identified by mass spectrum on the basis of their fragmentation patterns.

### Nucleic acid manipulations

The amino acid sequence of *Gaeumannomyces graminis *linoleate diol synthase (LDS) [17] was used to perform a BLASTp search of the *A. niger *N402 [18] genomic database (DSM food specialties, Delft, The Netherlands). Three putative dioxygenase genes (*ppoA*; GeneID: 4990997, *ppoC*; GeneID: 4985482 and *ppoD*; GeneID: 4979282) were identified that predicted proteins with high similarity to LDS. These genes were aligned to the *ppo *genes from *A. nidulans *and to the LDS from *G. graminis *and a phylogenetic tree was created using the ClustalW program http://www.ebi.ac.uk/clustalw. Pairwise scores between amino acids were represented as the number of identities and positives.

Primers to amplify fragments for complete gene (constructs containing promoter, gene and terminator) and disruption constructs were based upon the *A. niger *N402 genome sequence. These primers introduced restriction sites at either site of the amplified fragment during a PCR reaction (Table 3). *A. niger *genomic DNA was isolated using previously described techniques and used as the PCR template [19]. PCRs were carried out with AccuTaq LA™ DNA polymerase according to the manufacturer's protocol (Sigma) and the annealing temperature varied between 52°C and 60°C. Amplified PCR products were cloned into the pGEMTeasy vector (Promega, Madison, WI) and used to transform competent *Escherichia coli *DH5α. Positive clones containing the fragments for complete gene or disruption constructs were analyzed by restriction mapping and sequence comparisons to the NCBI genetic database using the tBLASTn algorithm http://www.ncbi.nlm.nih.gov.

**Table 3:** Primers used in this study

	Sequence 5' → 3'
*Constructs of complete genes*	
	
pMW012	
	
*ppoA*-dw	GAGGTGGGTCTTGTTTG
*ppoA*-up	GACAAACAGGGAGTTGC
pMW036	
	
*ppoD-dw*	GATTTCTTCCAGCTGGC
*ppoD-up*	GCTACAGCTACAGCTAC

*Disruption constructs*	
	
pMW051	
	
*ppoA3'-Nsi*I-dw	ATGCATGGTGGCAAACCAAGCC
*ppoA3'-Kpn*I-up	GGTACCGGTGAGGAGCACTACTTG
*ppoA5'-Hind*III-dw	AAGCTTATTTGTAGAGTCGAGG
*ppoA5'-Sph*I-up	GCATGCCATGCTTACCGTGAATG
pMW061	
	
*ppoD5'-Kpn*I-dw	GGTACCTTCCAGCTGGCATTGGTG
*ppoD5'-Bam*HI-up	GGATCCGTGCAGGGCCTTGAGCC
*ppoD3'-Sph*I-dw	GCATGCTGAAGCGCAACGTCTAAC
*ppoD3'-Hind*III-up	AAGCTTCAGCCCGTAGTTCTG

### Creation of disruption and complete gene constructs

Primers for fragments for disruption constructs were designed at the 5' and 3' flanking regions of predicted catalytic domains of PpoA, PpoC and PpoD. These catalytic domains were identified by ClustalW alignment of predicted PpoA, PpoC and PpoD to the LDS from *G. graminis *of which the catalytic domain has been identified [17]. Amino acids 202 to 883 for PpoA and aminoacids 224 to 1010 for PpoD were deleted. These contained for both PpoA and PpoD the distal (202; 265, respectively) and proximal (377; 444, respectively) His, and Tyr (374; 441, respectively) residues, essential for catalytic activity of PGS. Primers for complete genes were designed approximately 80 bp outside of the coding region.

Disruption constructs for *ppoA*, *ppoC *and *ppoD*, including the *argB *marker gene, were created as follows [20]. First, the 5' and 3' flanking regions were amplified by PCR introducing the indicated restriction sites (Table 3). The amplified products were digested from pGEMTeasy, separated on 0.8% agarose gel and isolated. The flanks were ligated into the pUC19 vector (Fermentas, Ontario, Canada) containing the *argB *cassette (pRV542) previously digested with the appropriate restriction enzymes resulting in the disruption constructs for *ppoA*, *ppoC *and *ppoD*. Disruption constructs were linearized by digestion with *Kpn*I/*Hind*III and used for *A. niger *transformations.

### *A. niger *transformations

Protoplasts were prepared from *A. niger *UU-A049.1 as described and transformed using polyethylene glycol [21]. Transformation of *A. niger *UU-A049.1 with *ppoA *and *ppoD *disruption constructs created transformants to ArginineB prototrophy with the catalytic domain of the corresponding gene product deleted. Three independent Aspergillus niger transformations did not result in the isolation of a *ppoC *disruptant and we were therefore not able to analyze this gene disruption. Transformants were purified by repeated streaking of conidia. Gene replacement and ectopic integration of the *argB *marker gene were checked by PCR and Southern analysis using internal fragments as probes.

### Probe construction and Southern analysis

Constructs of complete genes of *ppoA *and *ppoD *were digested with *Eco*RV and *Sph*I, respectively, yielding internal probes for the encoding region of the catalytic domain. Fragments were separated on an 0.8% agarose gel, isolated and randomly labeled with [α-^32^P]dCTP. This resulted in 1082 and 1146 bp fragments for *ppoA *and a 1241 bp fragment for *ppoD*. Chromosomal DNA of *A. niger *transformants was digested with the appropriate restriction enzymes. Hybridization with radioactive probes was done as described, except that washing of the filters was done at 65°C [22]. Positive transformants, lacking the signals from the internal probes on the Southernblot, were selected and used for further characterization.

### Phenotypic characterization of *A. niger *transformants

Characterization of *A. niger *transformants was performed on solid minimal medium containing 1% glucose and supplemented with or without 1 M NaCl and/or 0.01% H_2_O_2 _at 30°C and 42°C. Spots of 10000, 1000, 100 and 10 conidia were pipetted on each plate and incubated. Strains *A. niger *49.1 and *A. niger *N402 were used as wild type. Spore production studies were carried out on plates containing 25 mL solid minimal medium and 1% glucose [3]. For each plate a 5 mL top layer of cool melted 0.6% agar minimal medium and 1% glucose containing 10^7 ^conidia of the appropriate strain was added. In some cases 1.5% methanol or 1.5% methanol and 10 μg/mL linoleic acid were added to both agar layers. Cultures were incubated at 30°C. Cores of 16 mm diameter were removed from each plates and homogenized for 1 min in 3 mL sterile water supplemented with 0.01% Tween-80 to facilitate release of the hydrophobic conidia. Spores were counted by using a haemacytometer.

### *A. niger *microarray analysis

*A. niger *N402 was grown at 30°C as sandwiched cultures [23] in minimal medium [15] with 25 mM maltose or 25 mM D-xylose as carbon source. Zonal mycelial samples from 3 sandwich cultures were combined and used for RNA analysis. Mycelium was ground using a microdismembrator and RNA was extracted using TRIzol reagent (Invitrogen, Carlsbad, CA) according to the instructions of the manufacturer. RNA was purified using Nucleospin RNA clean up (Macherey-Nagel GmbH, Düren, Germany). Concentration of RNA was measured at λ_260_. Quality of the RNA was analyzed on an Agilent 2100 BioAnalyzer using the RNA6000 labchip kit (Agilent Technologies, Palo Alto, CA). Biotin-labeled antisense cRNA was generated by labeling 20 or 2 μg of total RNA with the BioArray High Yield RNA transcription labeling kit (ENZO) or the Affymetrix Eukaryotic One-Cycle Target Labeling and Control Reagent package, respectively. The quality of the cRNA was checked using the Agilent 2100 bioanalyzer. The labeled cRNA was hybridized to Affymetrix *A. niger *Genechips. Absolute values of expression were calculated from the scanned array using the Affymetrix GCOS software after an automated process of washing and staining. Microarray Suite Affymetrix v5.1 (Affymatrix Inc., Santa Clara, CA), Spotfire DecisionSite (Spotfire Inc, Somerville, MA), GeneData Expressionist Analyst V Pro 2.0.18 (GeneData, Basel, Switzerland) and the R statistical environment http://www.r-cran.org were used for data analyses.

## Abbreviations

17:0: margaric acid, heptadecanoic acid; 18:2: linoleic acid, 9*Z*,12*Z*-octadecadienoic acid; 20:4: arachidonic acid, 5*Z*,8*Z*,11*Z*,14*Z*-eicosatetraenoic acid; 8-HOD: 8-hydroxy octadecadienoic acid; 8-HOM: 8-hydroxy octadecamonoenoic acid; 9-HOD: 9-hydroxy octadecadienoic acid; 13-HOD: 13-hydroxy octadecadienoic acid; 10-HOD: 10-hydroxy octadecadienoic acid; 8,11-diHOD: 8,11-dihydroxy octadecadienoic acid; 5,8-diHOD: 5,8-dihydroxy octadecadienoic acid; PGS: prostaglandin synthase; LDS: linoleate diol synthase; SPE: solid phase extraction; TMS: trimethylsilyl.

## Authors' contributions

This work was performed as part of the PhD thesis for MWW. MWW carried out most experimentation. SICK performed the spore production studies. All experiments were supervised by RPV. All authors have contributed to the experimental and analytical design. MWW, RPV, JFGV (thesis advisor) and GAV (thesis advisor) wrote the manuscript. All authors have read and approved the final manuscript.
